# The Impact of Pulmonary Rehabilitation on Mental Health and Quality of Life in Patients With Chronic Obstructive Pulmonary Disease (COPD): A Narrative Review

**DOI:** 10.7759/cureus.49230

**Published:** 2023-11-22

**Authors:** Elaf M Almdabgy, Ali Qader, Albandari A Binjahlan, Alia M Alshalawi, Amani Albeladi, Weaam S Alharbi, Kholood A Almehmadi

**Affiliations:** 1 Department of Internal Medicine, King Faisal Hospital, Makkah, SAU; 2 Department of Internal Medicine, Salmaniya Medical Complex, Manama, BHR; 3 Department of Internal Medicine, Faculty of Medicine, King Abdulaziz University, Jeddah, SAU; 4 Department of Internal Medicine, King Abdulaziz Specialist Hospital, Taif, SAU; 5 Department of Internal Medicine, Umm Al-Qura University, Makkah, SAU

**Keywords:** respiratory disease, emphysema, pulmonary obstructive disease, meditation, multidisciplinary approach, personalized medicine

## Abstract

Chronic obstructive pulmonary disease (COPD) is a complex, prevalent, debilitating, and degenerative disease that affects a large population, and the treatment options for the patients are limited. Although progress has been made in COPD pathogenesis, etiology, and management, there is still an unmet need to develop novel therapies. COPD management has recently seen a focus on a multidisciplinary pulmonary rehabilitation approach to help patients manage the disease better. This review primarily focuses on the role of pulmonary rehabilitation as a novel therapeutic strategy for treating and managing COPD, which is known to decrease patients' quality of life. Disease management and the beneficial effects of pulmonary rehabilitation in COPD are discussed. Subsequently, different methods that are employed in pulmonary rehabilitation are examined, including oxygen therapy, exercise, meditation, and education, emphasizing how they can help patients better manage COPD. Pathophysiology and the effect of pulmonary rehabilitation on the cellular level, such as the release of perforins and Th_1_ and Th_17 _cytokines, are also explored. The link between exercise and meditation during pulmonary rehabilitation therapy, which promotes repairing affected organs, is emphasized. Future perspectives on personalized medicine and its use in conjunction with pulmonary rehabilitation are also outlined. In conclusion, pulmonary rehabilitation holds significant promise for the management of COPD by addressing the present limitations of treatment. However, further research is essential to overcome and optimize treatment strategies for COPD patients.

## Introduction and background

Chronic obstructive pulmonary disease (COPD) includes a wide range of lung diseases characterized by persistent respiratory symptoms such as shortness of breath, cough, mucus production, and exacerbations. These symptoms result from structural abnormalities in the airways, leading to bronchitis and bronchiolitis, and the air sacs, resulting in emphysema. These produce persistent, frequently worsening airflow obstruction [1]. Common symptoms of COPD include a reduction in airflow due to a combination of small airway disease (obstructive bronchiolitis) and parenchymal destruction (emphysema) [2]. This causes a decrease in patients' quality of life (QoL) through physical and mental illness [3,4]. Ischemic heart disease is the leading cause of death worldwide, while COPD ranks third after the former and neoplasm [5]. COPD also affects males and females differently as the incidence in men was 13/1000 person-years, and in females, it was found to be 6.1/1000 person-years. Additionally, its incidence is also higher in smokers [6]. In concurrence with the incidence, the mortality is also higher in males than females, and there is a definitive trend of increasing COPD after 45 years, irrespective of sex [7]. It is estimated that 300 million individuals worldwide suffer from COPD, with a high prevalence rate of 12.2%, and is known to have caused 3.2 million deaths in 2015 [8,9]. There are multiple risk factors for COPD, such as exposure to air pollutants, smoking, exposure to hazardous materials, a wide variety of allergens, and, most importantly, metabolic disorders such as obesity and physical inactivity [10].

Although significant advances have been made in the management of COPD, it poses a significant burden on patients as well as the healthcare system. COPD is known to affect the QoL through mental and physical illness [11]. QoL is an essential indicator of well-being, and COPD is known to impair QoL as affected individuals cannot freely socialize due to impairment of physical and mental health [12]. 

In this regard, pulmonary rehabilitation (PR), a multipronged approach to treating COPD, is gaining importance as it includes a plethora of approaches such as medical intervention, exercise, meditation, and change in lifestyle [3,13]. Several studies have shown that PR works at the cellular level, decreasing physical and mental stress and increasing patients' QoL [14,15]. At the cellular level, PR has been shown to reduce systemic inflammation. PR is crucial in improving the body's immune response, which is often compromised in individuals with COPD. Chronic inflammation in COPD can result in immune dysfunction, making patients more susceptible to infections, especially respiratory infections. Out of the many approaches to PR, exercise and meditation are quite crucial as they help in increasing overall mental as well as physical well-being [16,17]. 

Although through decades of research, the knowledge of managing COPD has increased, the burden of disease is still high. Our review thus aims to explore the multidisciplinary approach of PR on physical and mental health. By understanding and scrutinizing current literature on PR, we aim to explore how it can be used to understand COPD better and potentially formulate new treatment strategies. We hope to enhance the current knowledge and contribute to further research in the clinical management of COPD.

## Review

Methodology

An extensive literature review was done to explore how PR affects COPD. Scientific articles were selected based on their quality and emphasis on the role of PR in the pathogenesis and treatment of COPD. Our review involved surveying multiple databases, including PubMed/MEDLINE (Medical Literature Analysis and Retrieval System Online) and Web of Science. Specific keywords such as “Pulmonary rehabilitation in COPD,” “Quality of life in COPD,” “Pathophysiology of Pulmonary rehabilitation,” and similar related terminologies were used. Scientific articles obtained through these search terms had to undergo meticulous assessment and a critical evaluation. Relevant findings, as well as insights on how PR affects COPD, were taken into consideration. Studies with ample population size were considered for our review. Only studies published in the English language were considered. Our review focussed on studies published in the last 15 years, excluding one study published in 1999 to include the most recent up-to-date research. This has allowed the authors to present current and relevant information.

QoL of COPD patients

COPD patients usually suffer from multiple comorbidities like cardiovascular diseases, diabetes, and osteoporosis [18]. Additionally, they have other risk factors like smoking and low BMI, which further worsen their health-related quality of life (HRQL) [18]. An increase in QoL can be obtained by reducing exacerbation frequency, stopping smoking, managing body weight, and regular exercise [11]. As a result, instruments measuring HRQOL are often included in assessing COPD patients, completing the classical clinical parameters. A study found that impairment of HRQL is a significant point in managing chronic COPD [19]. Thus, the measurement of HRQL has become essential in judging the effectiveness of treatment in COPD. These factors, if taken into consideration, can not only lower healthcare costs but also improve the overall QoL of COPD patients. COPD is virtually incurable, as the patient's breathing capacity is reduced gradually. Only the symptoms of COPD can be managed, and it adversely affects HRQL [20]. Medical interventions, efficacy, and physical and mental assessment are the basis of HRQL measurement [21]. Several studies have shown the importance of HRQL in assessing the overall QoL of COPD patients [22,23]. One study found that the onset of COPD at an early age causes significant worsening of HRQL due to the early onset of COPD complications [21]. Similarly, another study in India found that the most common symptoms associated with low HRQL are chest pain and dyspnea [24]. Thus, it is of paramount importance to check the HRQL of patients and modify the treatment regimen of COPD patients so that the effect on the QoL of the patients is minimal.

Properties of PR and outcomes among COPD patients

One of the major therapeutic interventions in COPD patients pertains to PR, which is vital in managing COPD and is considered a cornerstone in treating COPD [13]. PR is a multipronged approach focusing on exercise, medical intervention, patient health education, social and psychological support, lifestyle changes, and meditation. These have been known to bring positive changes and improve the patient's overall QoL [3,13]. Several studies have suggested that PR can be given at different stages of treatment, such as hospitalization, follow-up, or during routine hospital visits [15,25]. One study showed significant improvement in clinical parameters when patients with severe respiratory impairment were given multidisciplinary comprehensive respiratory rehabilitation compared to the control group, which only received oxygen and medicines [26]. Similarly, another study showed that when patients received multidisciplinary PR, clinical parameters such as chest wall expansion, lung function, six-minute walking test (6MWT), and grip strength showed marked improvement, which also helped improve their QoL [4].

On the other hand, it has been known that diagnosis and clinical manifestation of COPD leads to a higher rate of anxiety and depression and can lead to severe respiratory disease and sometimes death [25,27]. Thus, screening COPD patients for depression and anxiety and their prompt treatment can help increase the QoL [28]. Another treatment strategy is to include daily exercise, as pouring evidence suggests that exercise decreases symptoms of anxiety and depression in COPD patients [29,30]. Mental illness and cognitive dysfunction are quite common in patients with COPD, and the associated symptoms include hypoxia, hypercapnia, anxiety, inflammation, and depression [31,32]. As the disease progresses, these symptoms lead to impairment of cognitive function, memory loss, poor health, and cognitive deterioration [33]. Thus, it is essential to have early intervention through pulmonary rehabilitation in COPD patients to increase the QoL, decrease disease flare-ups, and reduce the cost of healthcare.

Effects of PR on the cellular level and its pathophysiology 

As COPD is a multifactorial disease, several cellular-level changes occur [14]. These mechanisms include detrimental changes at the cellular level, including infiltration of inflammatory immune cells such as macrophages, neutrophils, and lymphocytes [34]. These cells damage the surrounding cells by secreting cell products such as perforins and initiating inflammatory immune response by releasing Th_1_ and Th_17_ cytokines [35,36]. CD8+ T killer cells also play an essential role in initiating COPD, as some studies have shown that its concentration is increased in the lung tissue [37]. However, these CD8+ T killer cells are known to secrete cytokines, which help initiate lung inflammation and cause the disease [38]. In contradiction, several studies have shown that the levels of CD8+ T killer cells are similar between COPD and the control population, or their levels are not increased in COPD [39,40]. Oxidative stress is more prominent in COPD patients through various mechanisms such as NF-κB stimulation, impaired antiprotease levels, damage of the DNA causing cellular senescence, and generation of autoantibodies [34].

These pathological changes cause the disease and exacerbate the symptoms but can be mitigated by PR. Methods of PR such as exercise, health education, and behavioral changes help in reducing the symptoms of COPD by changing the cellular physiology towards a normal profile, i.e., decreasing the inflammation, reducing expression of inflammatory molecules, chemokines, cytokines, and decreasing recruitment of immune cells towards the site of inflammation [41,42]. PR is also crucial in improving the body's immune response, which is often compromised in individuals with COPD. Chronic inflammation in COPD can result in immune dysfunction, making patients more susceptible to infections, especially respiratory infections. Exercise training, an integral part of PR, has been shown to enhance immune function. Exercise activates the NK cells and cytotoxic T cells, boosts the phagocytic capacity of macrophages, and stimulates the production of anti-inflammatory cytokines, thus effectively improving the immune response [34,39]. 

**Figure 1:**
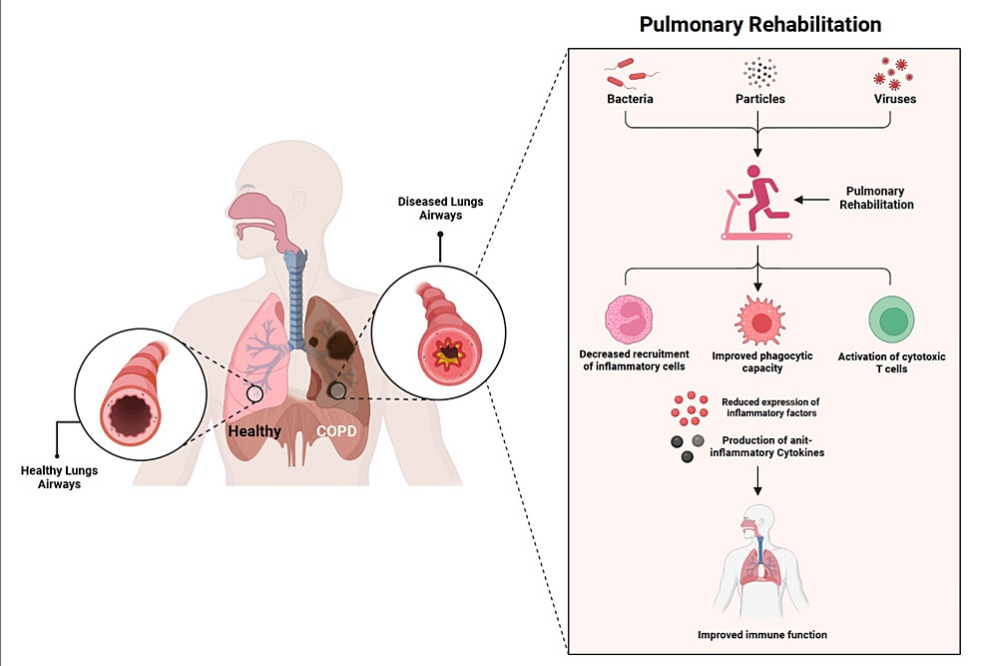
Demonstration of how pulmonary rehabilitation improves the immune function among patients with chronic obstructive pulmonary disease (COPD) Image credit: Authors

PR, a key component of COPD management, can help to mitigate these pathological changes. PR integrates exercise training, health education, and behavior changes, which together help to reduce the symptoms of COPD and improve QoL. Exercise training, an integral part of PR, has been shown to enhance immune function. It increases the activity of natural killer cells and cytotoxic T cells, boosts the phagocytic capacity of macrophages, and stimulates the production of anti-inflammatory cytokines, thus effectively improving the immune response [41,42]. The intricate mechanism of how cellular level changes occurs during PR of COPD patients is depicted in Figure 1.

Multidisciplinary approach to PR of COPD patients: role of exercise and meditation

PR is a fundamental component of standard extensive care for patients with COPD [26,43]. Although it is well known that PR of COPD patients increases their QoL and imparts physical and physiological benefits, it is still not used extensively worldwide [15,44]. In this regard, exercise training, education, emotional support, and nutritional support are the central tenets of this multidisciplinary therapeutic approach, all of which aim to enhance health, functional status, and QoL, including exercise maintenance in relation to one's health [16,43]. Neuroprotective effects of PR are also well established, and numerous studies have shown that PR can not only improve cognitive function but can also help in increasing the neurotransmitter release from the cerebral cortex, thus enhancing memory and cognition in a wide variety of populations [16,17,43]. Similarly, cognitive impairment brought on by COPD may account for variations in how individuals respond to PR interventions.

Nevertheless, concurrent cognitive impairment may limit PR's effectiveness [43]. For COPD patients, exercise training is a crucial component of PR. Pouring literature suggests that regular exercise can improve the function of the central nervous system and minimize the risk of cognitive impairment [32,45,46]. One study has shown that when exercise training is implemented, elderly COPD patients perform better on language fluency tests, implying that exercise can help increase cognitive power [32]. Interestingly, this study also found that the patients who underwent a 10-week exercise program, and after a year of follow-up, the researchers found that 39% of them kept up their regular exercise, while those who stopped showed cognitive deterioration [32]. Other studies have also found that exercise or meditation can help PR of COPD patients. A randomized clinical trial study by Wang et al. found that including the Tai Chi exercise program increased the 6MWT in patients and reduced their COPD Assessment Test (CAT) score [47]. Similarly, another double-blind controlled study found that pranayama (yoga breathing) as a measure of PR increased the 6WMT in COPD patients and helped increase their inspiratory capacity and air trapping [48]. Thus, it can be concluded that exercise and meditation as a part of PR can help increase the QOL of patients. 

Effects of PR on anxiety and depression among COPD patients

One study found that intervention through pulmonary rehabilitation can lead to improvement in anxiety and depression among COPD patients, although this study did not include COPD patients suffering from severe depression or anxiety [49]. However, another large-scale study of 734 patients found that including PR in COPD patients through exercise, education, depression, and anxiety exercise was able to significantly reduce the level of chronic depression and anxiety [50]. Another study by Tselebis et al. also found that a PR program was able to reduce the levels of depression and anxiety significantly in COPD patients [51]. Current literature suggests that including a PR program can reduce the levels of normal and chronic depression and anxiety among COPD patients.

Personalized medicine and PR in COPD

The literature suggests that COPD is not a simple or single disease but can be defined as a collection of different abnormalities that are controlled by a plethora of molecular processes and abnormalities [52]. Advancement in disease etiology and understanding of the disease can help researchers and clinicians define different subtypes of COPD. Precision medicine, which is known to target specific blood markers in COPD, can help alleviate the disease [53]. If used in conjunction, precision medicine targets specific markers, and PR can help lower the disease burden of COPD [54]. For example, in predicting the potential efficacy of incorporating inhaled corticosteroids alongside routine bronchodilator therapy to reduce the frequency of exacerbations at an individual level. A suggested threshold of 100 eosinophils/μL has been proposed, below which the administration of corticosteroids is unlikely to yield substantial clinical benefits [53]. This can be achieved through programs that are individually tailored to the clinical symptoms and disease type of COPD of individual patients. Intervention would be complex and should integrate physical, physiological, and psychological outcomes and instill long-term adherence to the program [55]. 

## Conclusions

PR offers an exciting potential to not only manage COPD but also help in increasing the QoL of COPD patients. PR has a unique ability to help COPD patients through a multidisciplinary, multipronged approach. PR works by integrating treatment with exercise, meditation, and education of patients and thus highlights the divergent ways through which it addresses the complex process of COPD. However, several challenges remain, such as optimization of exercise programs, meditation, and education so that long-term adherence and significant clinical benefits are observed. Thus, advancement in PR with the implementation of different programs, completion of more randomized clinical trials to test their efficacy, and combination with personalized medicine and approaches will help in better management of COPD in the future. Thus, through harnessing the potential of PR and personalized medicine, a future can be visualized where COPD patients have improved outcomes, greater QoL, and less mental and physical stress.
